# Optical Anisotropy of Porphyrin Nanocrystals Modified by the Electrochemical Dissolution

**DOI:** 10.3390/molecules27228010

**Published:** 2022-11-18

**Authors:** Rossella Yivlialin, Claudia Filoni, Francesco Goto, Alberto Calloni, Lamberto Duò, Franco Ciccacci, Gianlorenzo Bussetti

**Affiliations:** Department of Physics, Politecnico di Milano, p.zza Leonardo da Vinci 32, 20133 Milano, Italy

**Keywords:** reflectance spectroscopy, porphyrin, dissolution, organic crystals, cyclic voltammetry, AFM

## Abstract

Reflectance anisotropy spectroscopy (RAS) coupled to an electrochemical cell represents a powerful tool to correlate changes in the surface optical anisotropy to changes in the electrochemical currents related to electrochemical reactions. The high sensitivity of RAS in the range of the absorption bands of organic systems, such as porphyrins, allows us to directly correlate the variations of the optical anisotropy signal to modifications in the solid-state aggregation of the porphyrin molecules. By combining in situ RAS to electrochemical techniques, we studied the case of vacuum-deposited porphyrin nanocrystals, which have been recently observed dissolving through electrochemical oxidation in diluted sulfuric acid. Specifically, we could identify the first stages of the morphological modifications of the nanocrystals, which we could attribute to the single-electron transfers involved in the oxidation reaction; in this sense, the simultaneous variation of the optical anisotropy with the electron transfer acts as a precursor of the dissolution process of porphyrin nanocrystals.

## 1. Introduction

Reflectance anisotropy spectroscopy (RAS) has been widely exploited in the past in order to investigate the physical–chemical properties of organic systems featuring different solid phase aggregates and prepared using different growth techniques (Langmuir–Blodgett and Langmuir–Schaefer methods [1,2], physical vapor deposition [3,4], etc.). More specifically, RAS offers the possibility of studying systems that exhibit anisotropies (down to a signal intensity of 10^−6^ [5]) related to the electronic or morphological characteristics of the organic layer, even if it is grown onto an isotropic substrate such as graphite [6]. For instance, this optical spectroscopy has been successfully used to characterize complex 3D nanoarchitectures based on porphyrins layers in order to understand the mechanisms governing their interaction with the environment (i.e., vacuum or vapors), in view of their implementation in organic-based devices and sensors [7,8,9]. One of the advantages of RAS is the versatility of its experimental configuration, which allows the investigation, in situ, of systems working in different experimental conditions (e.g., UHV, ambient, liquid and gas) [10]. Among the possible environments, liquid media have drawn increasing attention in recent decades, given the increasing interest among the scientific community in the physical processes and chemical/electrochemical reactions occurring specifically at the solid/liquid interfaces [11,12], with the ultimate goal of reproducing or mimicking biological systems. In this respect, successful examples of the application of RAS to inorganic/organic systems studied in an electrochemical cell are the works of Mazine [13], Smith [14], Goletti [15], Barati [16] and Weightman [17]. These works demonstrate that changes in the surface optical anisotropies are strongly correlated with changes in the electrochemical current and that RAS can elucidate details on the atomic and molecular interactions associated with the electrochemical processes.

In the case of porphyrin layers, many studies have focused on the interface using acidic solutions. The morphological/optical evolution of porphyrin layers grown on metal electrodes has been investigated, for instance, by in situ electrochemical atomic force microscopy (EC-AFM) [18,19] and diffuse reflectance spectroscopy, and also *in operando* conditions, i.e., during the activation of electrochemical processes, typically by using cyclic voltammetry (CV) [20]. In particular, some of the authors of the present work recently investigated the particular phenomenon of the dissolution of porphyrin nanocrystals occurring during CVs in acidic solutions [20,21]. There, the possibility of gradually removing porphyrin layers by continuously sweeping the applied EC potential was observed by in situ EC-AFM. However, it was not possible to determine the exact onset of that process by only looking at the morphological evolution of the samples.

In this work, we exploit the coupling between RAS and an EC cell as a powerful tool to precisely determine the early stage of the dissolution process. In fact, by means of the in situ monitoring of the optical anisotropy, we can correlate the oxidation of the porphyrin films to the structural/morphological modifications of the 3D nanocrystals. RAS is used for the direct identification of the EC potential that instantaneously activates modifications in the aggregation state of the molecules in the film, operating as a driving force for the dissolution of the porphyrin nanocrystals.

For this work, a thick layer of porphyrins was vacuum-deposited on a graphite substrate, resulting in the formation of nanocrystals on the surface. This system is well-suited to both RAS and electrochemical techniques for two main reasons: (i) porphyrin nanocrystals in freshly grown samples exhibit a clear anisotropy signal in the Soret-band spectral region [22,23]; (ii) graphite covered with porphyrins behaves as a good working electrode for the electrochemical activities, showing well-defined CV curves [18]. We investigate the oxidation process and the optical/morphological evolution of three types of films, featuring porphyrins with nominally different molecular symmetries: free-base tetraphenylporphyrins (H_2_TPP) [24], zinc tetraphenylporphyrins (ZnTPP) [25,26] and iron(tetraphenylporphyrinato) chloride [Fe(TPP)Cl] [27]. These molecules differ one from another in the composition of the inner tetrapyrrolic ring, i.e., due to the presence in the middle of the ring of, respectively, no metal atom, a Zn atom and a Cl-coordinated Fe atom, which potentially influence inter-molecular arrangements and layouts of porphyrin nanocrystals.

## 2. Results and Discussion

**Figure 1:**
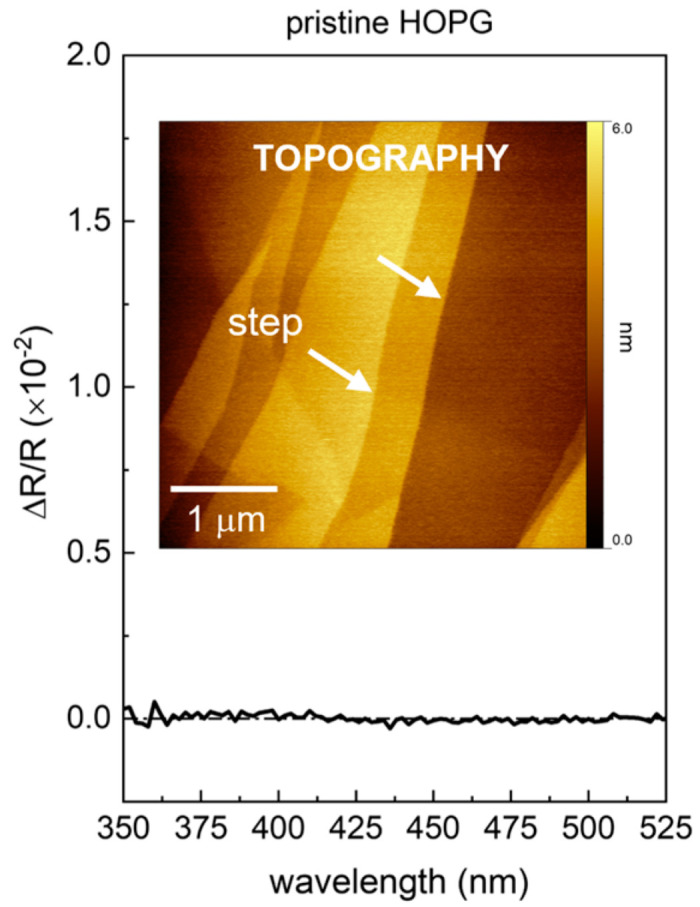
RAS spectrum in the 350–525 nm range of the bare HOPG substrate, acquired in air. The absence of any RAS feature testifies the optical isotropy of HOPG. The AFM image in the inset shows the topography of the HOPG surface in air condition; the white arrows indicate some steps on the surface.

Figure 1 shows the morphology and RAS spectrum of the bare HOPG substrate in air before the vacuum deposition of porphyrin films.

The AFM image shows the typical steps, only a few nm in height, created by the exfoliation of the HOPG surface [28]. The RAS spectrum was acquired as a reference for the subsequent measurement of the porphyrin films, and shows a flat isotropic signal, as expected from the hexagonal symmetry of the HOPG crystal. In the following, we first discuss those samples with a comparable RAS peak line shape: ZnTPP film and H_2_TPP films, the latter with a higher peak intensity. Lastly, we consider a Fe(TPP)Cl film, characterized by a different RAS signal.

### 2.1. ZnTPP Film

**Figure 2:**
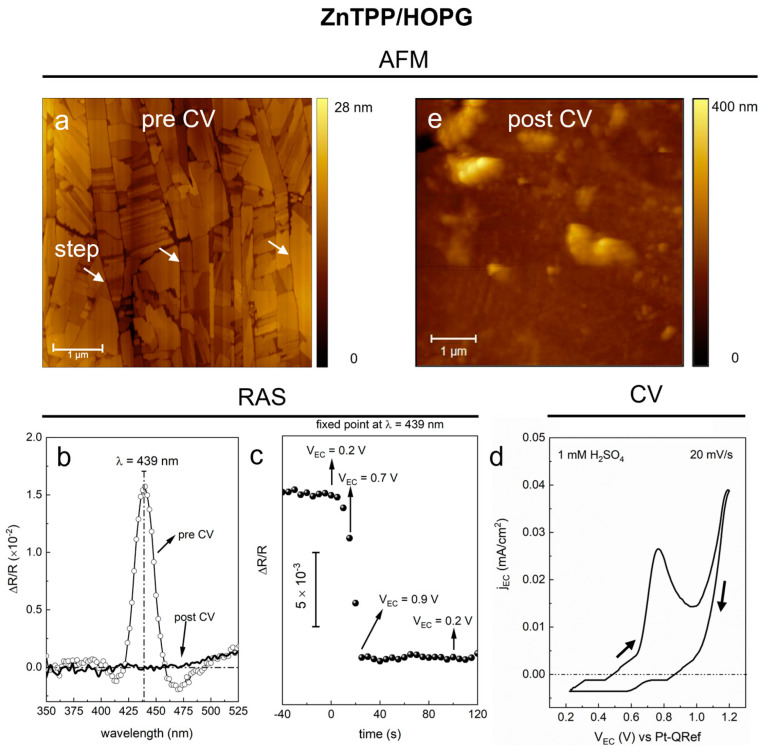
(**a**) (5 × 5) µm^2^ topographic image of a 6 nm thick ZnTPP/HOPG sample; white arrows points at some steps of the HOPG substrate. The AFM image was acquired ex situ on an as-deposited sample. (**b**) RAS spectra in the 350–525 nm range, acquired when the sample is in the EC cell filled with a 1 mM H_2_SO_4_ solution. Spectra acquired before (line + symbol) and after (solid line) the CV are reported. (**c**) Real-time RAS monitoring of the Soret-related band intensity (λ = 439 nm) during CV in a 1 mM H_2_SO_4_ solution. The time axis is rescaled to show all the time range covered by the sweep of the EC potentials in the CV; time = 0 s corresponds to the starting point of the CV (V_EC_ = 0.2 V); spectral points are collected every 5 s. The scale of the y-axis is showed by a vertical bar. Arrows and labels indicate the EC potentials reached during the CV. (**d**) Voltammogram obtained during the CV in a 1 mM H_2_SO_4_ solution; the potential is swept within the 0.2–1.2 V range, from anodic to cathodic regime (see the black arrows); sweep rate = 20 mV/s; step = 1 mV. (**e**) (5 × 5) µm^2^ topographic image of the ZnTPP/HOPG sample, acquired ex situ after the EC treatment in a 1 mM H_2_SO_4_ solution.

After the deposition of the ZnTPP film, the morphology of the sample was checked by AFM in air, before the immersion in the electrolyte. A representative scan is shown in Figure 2a.

For the ZnTPP film, we observe regular 3D porphyrin nanocrystals (about 20 nm high), with sharp edges and angles of almost 90°, laying on a 2D wetting layer [29]. For thick films, the topographic AFM signal gives evidence only of the 3D phase of the sample, due to the extension and high density of the nanocrystals, which hide the 2D wetting layer on the surface.

As extensively discussed by the authors in previous works [22,29], the anisotropy of this kind of samples is maximized when the direction of graphite exfoliation is aligned with the α(β) direction, meaning that ZnTPP nanocrystals are preferentially oriented along the graphite steps. A statistical analysis on all the collected AFM images seems to further confirm this scenario: the image shown in panel *a*, for instance, shows the nanocrystals clearly laying on the graphite terraces, with most of the edges parallel to the steps (see the vertical lines indicated by white arrows).

Since we are interested in the variation of the anisotropy signal when the sample undergoes an electrochemical oxidation in sulfuric acid, the RAS signal was measured on the sample in pristine conditions, as soon as was immersed in the acidic solution and before running the CV. The initial optical anisotropy of the ZnTPP film is clearly visible in the RAS signal (labeled as “pre CV”) of Figure 2b, where a main positive RAS peak is observed, centered at 439 nm (FWHM = 20 nm), with an intensity of about 1.6 × 10^−2^ with respect to the zero line.

This anisotropy arises from the molecular packing in the 3D nanocrystals, since isolated ZnTPP molecules are centrosymmetric, meaning they do not contribute to the overall anisotropy of the sample [22]. A closer inspection of the RAS signal highlights the presence of a negative feature placed at about 470 nm. The overall “pre CV” RAS signal, collected in the Soret-band region, shows the characteristic “derivative-like” shape reported and discussed in the literature [7]. This particular signal behavior directly arises from the superposition of different optical contributions, due to the complex stratified structure of the thin porphyrin films [30,31].

Changes in the RAS signal correlated to chemical processes involving the 3D nanocrystals can be monitored in real-time by following the RAS signal intensity at a fixed wavelength (namely, the one related to the Soret-band at 439 nm) while running the CV. The obtained results are reported in Figure 2c,d.

By comparing panels c and d, we observe that the intensity of the anisotropy signal drastically drops (by about ¼ of the initial value) as soon as the EC potential approaches the value of 0.7 V, which corresponds to the onset of the main peak shown in the voltammogram. This peak indicates the presence of an oxidative process, where ZnTPP molecules form radical cation species (V_EC_ = 0.8 V) [20], which anticipates the oxygen evolution reaction (OER) at the sample surface (onset at about V_EC_ = 1.0 V).

Once the V_EC_ = 0.9 V threshold is reached, i.e., after about 25 s from the start of the CV, the RAS anisotropy signal stabilizes around a mean value of zero until the CV is completed (final V_EC_ = 0.2 V, see panel *c*), with an overall variation of about 1 × 10^−2^ from the initial value. The disappearance of the optical anisotropy signal from the sample due to the oxidative process is confirmed by the extended RAS spectrum acquired at the end of the CV, as shown in Figure 2b (the spectrum labeled as “post CV”). There, the contrast between the zero signal of the “post CV” spectrum and the sharp peak in the “pre CV” spectrum points out that the EC treatment makes the ZnTPP film, on the macro-scale, optically isotropic in the Soret-band region. As discussed above, the variation of the optical anisotropy of the sample must be related to changes in the morphology or to some removal of the organic layer. In this case, it is likely related to modifications of the 3D nanocrystals. It was already demonstrated by the authors, indeed, that ZnTPP porphyrin nanocrystals undergo some dissolution phenomena during the CVs in acidic solutions [20], while they are stable in static conditions (i.e., when ZnTPP molecules are immersed in the acidic solution, with no EC potential applied) [21].

The morphology shown in Figure 2e confirms the complete loss of crystallinity in the ZnTPP 3D phase of the EC treated sample. The flat paving of the surface created by the dense distribution of regular and oriented nanocrystals, shown in panel a, is replaced by the presence of different agglomerates with irregular shapes and undefined orientations; in addition, graphite steps cannot be distinguished consistently in the case of an amorphous layer covering the substrate [32,33].

### 2.2. H_2_TPP Film

**Figure 3:**
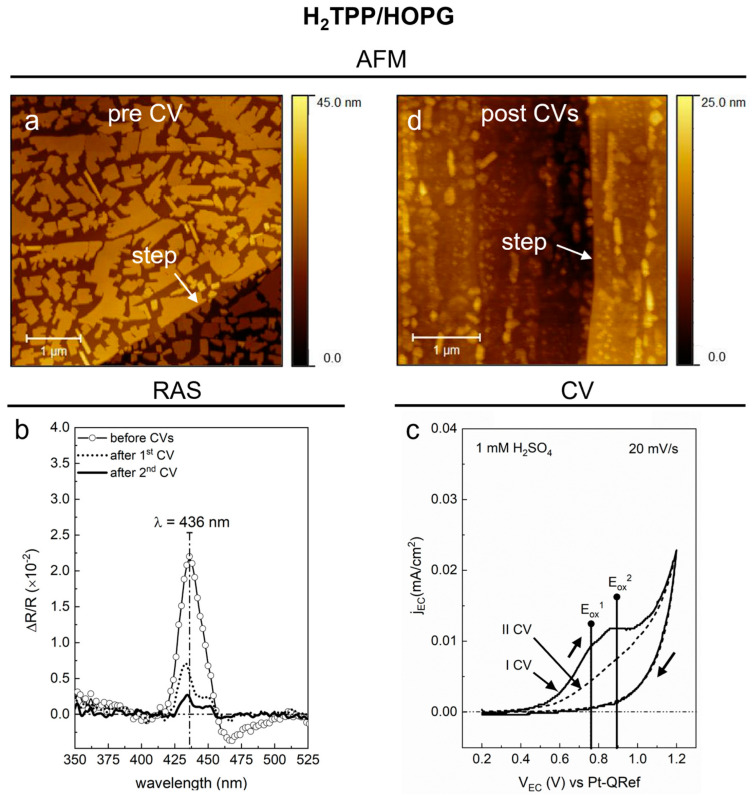
(**a**) (5 × 5) µm^2^ topographic image of the 6 nm thick H_2_TPP/HOPG sample; the white arrow points at a step of the HOPG substrate. The AFM image was acquired ex situ on the as-deposited sample. (**b**) RAS spectra in the 350–525 nm range, acquired when the sample is in the EC cell filled with a 1 mM H_2_SO_4_ solution. A comparison between three spectra is reported: one acquired before the CVs (line + symbol), a second one after the first CV (dotted line), and a third one after the second CV (solid line). (**c**) The voltammograms obtained during the two consecutive CVs (labeled as I and II) in a 1 mM H_2_SO_4_ solution are shown; range: (0.2–1.2) V, from anodic to cathodic regime; sweep rate = 20 mV/s; step = 1 mV. (**d**) (3.5 × 3.5) µm^2^ topographic image of the H_2_TPP/HOPG sample, acquired ex situ after the two EC treatments in a 1 mM H_2_SO_4_ solution; the white arrow points at one of the steps of the HOPG substrate.

A behavior similar to that of ZnTPP was observed for the free base-TPP film. The initial morphology of the H_2_TPP/HOPG film, observed ex situ on the as-deposited sample, is reported in Figure 3a.

In this figure, 3D porphyrin nanocrystals are shown to be covering the HOPG. In comparison with ZnTPP in Figure 2, nanocrystals look more dendritic, with a lower (about half) surface to volume ratio, calculated by evaluating the bearing area (volume), i.e., the area (volume) of the portion of nanocrystals getting out of the basal plane of the image; the edges and angles are sharp and well-defined, as are the ones observed on the pristine ZnTPP sample. The preferential orientation of the nanocrystals in the exfoliation direction of the HOPG substrate is also confirmed in this case by the RAS azimuthal analysis [29]. The maximum RAS signal is shown in Figure 3b (line + symbol) before the EC treatment.

By comparing it with the “pre CV” spectrum of the ZnTPP sample, we note that the Soret-band shows a similar line shape (FWHM = 20 nm), a blue-shifted spectral position (λ = 436 nm) and a higher intensity of about 2.2 × 10^−2^. These small differences in the RAS signal can be reasonably attributed to the different morphology of ZnTPP and H_2_TPP nanocrystals.

As for the ZnTPP film, the anisotropy of the thick H_2_TPP film originates from the 3D phase of the sample. We point out that, at variance from the ZnTPP case, also the H_2_TPP 2D phase shows an intrinsic anisotropy (a RAS feature at about 430 nm with an intensity in the 10^−3^), created by the alignment the NH-atoms inside the macrocycle of the single molecules along the same direction, as extensively demonstrated by some of the authors [6,22]. However, in the RAS spectrum of Figure 3b, the anisotropy signal coming from the 2D phase is completely hidden by the much more intense signal from the 3D phase (one order of magnitude larger).

The intensity of the Soret-band starts to reduce (evolution not reported here) when the EC potential reaches the value V_EC_ = 0.6 V, during a first CV in a 1 mM H_2_SO_4_ solution (see “I CV” in Figure 3c). Similar to the ZnTPP case, the anisotropy of the H_2_TPP sample is affected by the oxidative process of the molecular film, whose onset is placed at 0.6 V, represented by the appearance of two small anodic features at about V_EC_ = 0.75 V and V_EC_ = 0.9 V in the CV [18]. These features are related to two one-electron oxidation processes (usually called E_ox_^1^, E_ox_^2^) of the H_2_TPP molecule which involve the porphine macrocycle and mostly the activity of the inner N atoms [34].

Differently from ZnTPP, the anisotropy of the H_2_TPP film is not completely canceled by the EC treatment. In fact, the RAS signal acquired at the end of the first CV (dotted-line, panel b) shows a not-null anisotropy signal where the two main features of the pristine samples are still visible, although with an intensity of about 1/3 of the initial value. A similar line shape is observed, albeit with an even less intense anisotropy signal, after a second CV (full line, panel b); however, the voltammogram of the second CV (“II CV” in panel *c*) does not clearly show the two one-electron oxidation features observed in the first CV. This behavior suggests that the outer porphyrin layers of the sample, more exposed to the acidic solution, have been fully oxidized or dissolved by the first EC treatment, which modified, without completely removing, the total initial anisotropy of the molecular film. The residual lower anisotropy signal, observed both after the first (dotted-line spectrum) and the second EC treatment (solid line spectrum), indicates that while some H_2_TPP layers may have been dissolved during the oxidation, a few layers persist on the HOPG surface [20]. It is worth mentioning that the RAS signal acquired after the second CV shows a shoulder at about 430 nm, with an intensity of about 1.3 × 10^−3^. This feature is similar to the anisotropy signal related to the 2D phase observed on thin H_2_TPP/HOPG samples, supporting the idea that the EC oxidation causes the dissolution of the uppermost porphyrin layers and the modification of the nanocrystals, making the original thick film closer to a thin H_2_TPP film.

An additional note is that the H_2_TPP nanocrystals, differently from the ZnTPP ones, are sensitive to the acidic media and undergo etch-pitting phenomena with time, even when they are immersed in a H_2_SO_4_ solution in static conditions. In the work of ref. [21], it was observed that the etch-pits created on the nanocrystal surface are well-aligned to the crystal edges. Even though the etch-pitting in static conditions is not equivalent to an EC dissolution and is not the focus of the present work, the information gained in our previous work can nevertheless give us an indication of the preferential crystallographic directions involved in the EC dissolution process of the H_2_TPP nanocrystals. We speculate that even the dissolution caused by the EC oxidation of the sample starts from the edges of the nanocrystals, thus preserving a certain degree of order in the organic film that we see in the residual anisotropy signals measured after the CV.

The final morphology of the sample, revealed by ex situ AFM, is shown in Figure 3d. As for the ZnTPP 3D phase, the H_2_TPP 3D phase after the EC treatment looks quite changed compared to the pristine condition (panel a) and in agreement with previous observations of the morphology of H_2_TPP films treated in different kind of acid solutions [18,19]. Differently from the ZnTPP case in Figure 2, here, some form of crystallinity appears to have been preserved and some regular shapes, resembling nanocrystals, are still recognizable; in addition, the prevailing orientation of these 3D structures is still aligned along the HOPG steps. This kind of morphology supports the interpretation of the spectra measured by RAS (Figure 3b), where the residual final anisotropy looks like the convolution of a signal coming from the 2D phase and another one from an electrochemically modified 3D phase.

### 2.3. Fe(TPP)Cl Film

**Figure 4:**
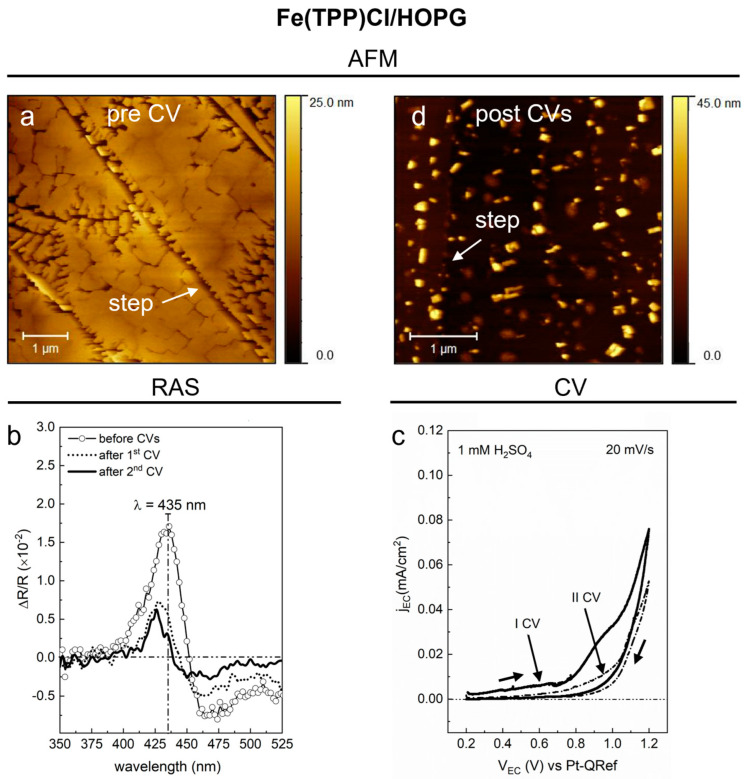
(**a**) (5 × 5) µm^2^ topographic image of the 6 nm thick Fe(TPP)Cl/HOPG sample; the white arrow points at one of the HOPG substrate steps. The AFM image was acquired ex situ on the as-deposited sample. (**b**) RAS spectra in the 350–525 nm range, acquired with the sample in the EC cell filled by a 1 mM H_2_SO_4_ solution. A comparison between three spectra is reported: one acquired before the CV (line + symbol), a second one after the first CV (dotted line), and a third one after the second CV (solid line). (**c**) Voltammograms obtained during the two consecutive CV (labeled as I and II) in a 1 mM H_2_SO_4_ solution; range: (0.2–1.2) V, from anodic to cathodic regime; sweep rate = 20 mV/s; step = 1 mV. (**d**) (3.5 × 3.5) µm^2^ topographic image of the Fe(TPP)Cl/HOPG sample acquired ex situ after two EC treatments in a 1 mM H_2_SO_4_ solution; the white arrow points at one of the steps of the HOPG substrate.

An example metal-porphyrin with a different molecular symmetry with respect to ZnTPP is represented by Fe(TPP)Cl, the Cl atom being an axial ligand for the Fe atom placed in the middle of the tetrapyrrolic ring. The morphology of the Fe(TPP)Cl/HOPG sample in pristine condition is reported in Figure 4a.

In this case, nanocrystals present different shapes; some of them look like the regular blocks observed on ZnTPP and H_2_TPP films, while some others have an irregular and dendritic geometry; again, most of the edges of these structures are aligned to the graphite steps.

The anisotropy signal measured on this sample is labeled as “before CVs” in Figure 4b. As well as for the ZnTPP sample, even for the Fe(TPP)Cl sample, we expect that the main optical anisotropy feature originates from the 3D molecular aggregates. The Soret-band is centered at 435 nm, with an intensity of about 1.7 × 10^−2^ with respect to the zero line. However, the line shape is more structured than the one observed on the previous samples, presenting a shoulder at 407 nm and a deeper valley at about 470 nm, just before the upcoming Q-bands (not visible in Figure 4b). Interestingly, this result agrees well with the optical spectra measured by Kadish and coworkers on mixed dimeric-monomeric Fe(TPP)Cl systems, where dimers are represented by two porphyrins in the (Fe^III^TPP)_2_O form, held together by a single O ion bridging the metal atoms [35,36]. In our Fe(TPP)Cl sample, the presence of dimeric-monomeric species could be the reason also for the peculiar mixed morphology observed by AFM (see panel a).

The initial RAS signal starts to change during the first CV in sulfuric acid when the EC potential reaches the value of 0.8 V, as we verified by monitoring the Soret-band intensity during the EC process (data not reported here). The voltammogram in panel *c* shows an anodic feature at about V_EC_ = 0.9 V, just before the OER potential, which corresponds to the single-electron oxidation Fe(III) → Fe(IV) of the middle atom in the porphyrin molecule [36,37]. We speculate that this oxidation proceeds in parallel with a destruction of the porphyrin dimers, leading to variations in the morphology of the Fe(TPP)Cl nanocrystals, as we can argue by looking at the anisotropy signal in the RAS spectrum of panel b labeled “after 1st CV”. There, both the intensity and the line shape of the Soret-band were strongly changed, pointing out that a quantitative and qualitative modification of the organic film occurred.

No significant variations of the anisotropy signal, instead, are observed after a second CV (“after 2nd CV” spectrum in panel b), as one could expect by looking at the corresponding featureless voltammogram (dash-dotted line in panel c).

The final morphology of the Fe(TPP)Cl film, as observed by AFM, is reported in Figure 4d. This topographic image shows that the 3D phase was effectively changed from the one we observed in pristine condition. Now, narrower and higher porphyrin aggregates, with almost regular edges, cover the HOPG steps and terraces, and, more interestingly, it is no longer possible to discern the dendritic structures, such as the ones clearly observed in panel a. All these results suggest that the Fe(TPP)Cl dimeric-monomeric system undergoes a structural modification during the oxidation process in sulfuric acid, which implies the disruption of the 3D dendritic phase and the consequent modification of the morphology and optical anisotropy of the porphyrin film.

## 3. Materials and Methods

### 3.1. Sample and Solution Preparation

**Scheme 1:**
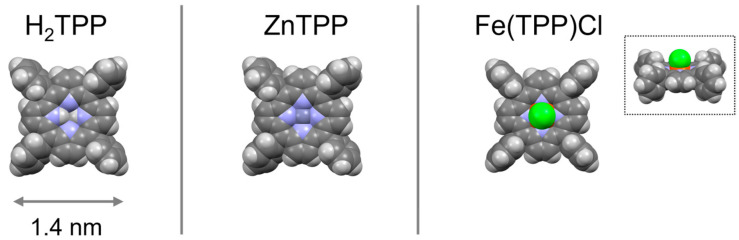
Sketch of three different porphyrins used in the experiments: dark grey balls = C-atoms; light grey balls = H-atoms; violet balls = N-atoms; dark violet ball = Zn-atom; red ball = Fe-atom; green ball = Cl-atom; the average molecular size is indicated at the bottom. In the Fe(TPP)Cl molecule, the Cl-atom is placed axially on top of the Fe-atom; a side view of the molecule is reported in the inset.

Samples were present in films with a nominal thickness of 6 nm featuring three kinds of tetraphenylporphyrin (TPP) molecules: H_2_TPP, ZnTPP and Fe(TPP)Cl (see Scheme 1).

The organic films were deposited in vacuum on highly oriented pyrolytic graphite (HOPG, by Optigraph GmbH, with a mosaic spread of 0.4 degree to ensure a high reflectivity useful for the optical spectroscopy characterization). Before each molecular deposition, HOPG was exfoliated using an adhesive tape always along the same direction, in order to create a preferential orientation for the HOPG steps, thus orienting the porphyrin nanocrystals and ensuring a special direction for the alignment of the RAS apparatus.

The film were grown in an organic molecular beam epitaxy (OMBE) chamber, equipped with heated crucibles for molecular evaporation [38]. The sublimation rate was measured by means of a quartz microbalance and kept at 1 Å/min, corresponding to a sublimation temperature in the range of 260–285 °C for all the molecules. The substrate was kept at RT during the deposition. In these experimental conditions, porphyrins form nanocrystals by following a “layer-plus-island” growth process (i.e., Stranski–Krastanov growth) [23].

The electrochemical solution consists of 1 mM H_2_SO_4_ (pH = 3), prepared by diluting in water concentrated H_2_SO_4_ (95–97% *w*/*w*, Merck); the solution was degassed by bubbling pure Ar in a separator funnel for some hours before each experiment.

### 3.2. RAS Apparatus and EC Cell

**Figure 5:**
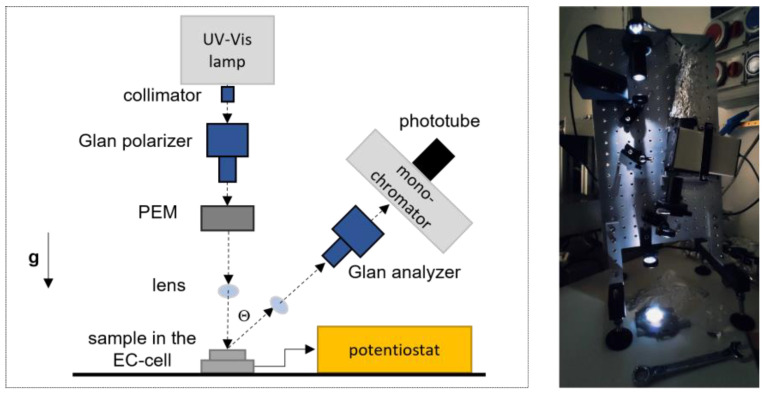
Sketch and picture of the experimental set-up used to couple RAS with the electrochemical measurements. The light beam illuminates the sample from the top-aperture of the EC cell, placed on the table. Reflected light is collected within an angle of about 4° from the impinging beam direction (see the main text for details).

The experimental set-up which combines RAS technique to the EC cell is reported in Figure 5.

A high-sensitivity homemade RAS apparatus [5] with a light spot diameter of about 5 mm was used to collect the spectra. The light coming from a Xe arc lamp was linearly polarized (α-direction) by a Glan–Thompson optical system and passed through a Photo-Elastic Modulator (PEM), which rotates the light polarization along two mutually orthogonal directions (α and β) at a double frequency with respect to the resonance frequency of 50 kHz. The light beam was properly focused on the sample by a system of lenses and reflected within an angle of few degrees (Θ). Along the reflected light-path, after a second system of lenses, a second Glan polarizer (called analyzer) linearly polarized the light coming from the sample; then, a monochromator and a photomultiplier detector followed.

The RAS signal is defined as the difference in the reflectivity between α and β directions ($R_{\alpha },R_{\beta }$) normalized by the total reflectivity ($\bar{R}$):

(1) $$\frac{\Delta R}{\bar{R}}=2\frac{R_{\alpha }\left(\omega \right)-R_{\beta }\left(\omega \right)}{R_{\alpha }\left(\omega \right)+R_{\beta }\left(\omega \right)}\;$$

The anisotropy of the sample is detected by a phase-sensitive lock-in amplifier from EG&G (San Francisco, CA, USA) measuring the signal modulation $\Delta R=R_{\alpha }-R_{\beta }$ at the frequency provided by the PEM oscillation.

In the case of thin organic layers, where a complex stratified structure of molecules characterize the film, the reflected light is generally termed as “reflectance”, in place of “reflectivity” [39].

It is worth mentioning that the alignment of the sample with respect to the α and β directions of PEM is, *a priori*, unknown; an azimuthal analysis is required to find the maximum of the RAS signal. For the molecular samples analyzed in this work, the maximum value of the anisotropy (in modulus) is reached when the direction of graphite exfoliation is aligned along the *α* or *β* direction of PEM (within 10°) [22,29]. RAS measurements were collected in the 330–650 nm wavelength (λ) range; however, in order to focus the discussion on the main porphyrin optical transition, only a shorter range centered on the Soret-transition band at about 436 nm is reported in the text. The evolution of the anisotropy of the samples, which occurs during the EC treatments in solution, was observed by monitoring the variation of the Soret-band intensity while applying different EC potentials to the samples. For this purpose, RAS measurements at fixed λ, corresponding to the spectral position of the Soret-band, were performed by collecting a spectral point every 5 s, according to the sweeping rate used to change the EC potentials during the CV.

To combine the optical spectroscopy with the electrochemistry techniques, in this specific set-up, the RAS apparatus is vertically positioned above the sample. The latter is located inside a home-made three-electrodes EC cell, made of Teflon^®^, with a top aperture for the light beam. The EC cell is filled by about 1 mL of solution, and the area of the sample surface exposed to the solution is about 20 mm^2^.

The sample represents the working electrode (WE), while two platinum wires are used, respectively, as a counter electrode (CE) and a reference electrode (RE); the latter is a Pt quasi-reference (Pt-QRef) with a stable (within few mV) potential shift of +740 mV vs. a standard hydrogen electrode (SHE) when immersed in acid solution. The EC cell is connected to a potentiostat (PalmSens4) to perform the CV. By running the CV, a linear ramp of potentials is applied to the WE, in the 0.2–1.2 V range, with a sweep rate of 20 mV/s.

### 3.3. Atomic Force Microscopy

AFM characterizations were collected using a commercial scanning probe microscope (5500 by Keysight Technology, Santa Rosa, CA, USA). The images were acquired in tapping-mode, with silicon tips from NanoSensors, Neuchatel, Switzerland (cantilever force constant: 10–130 N/m; ν_0_ = 223 kHz) and typical scan rates of 1.2 line·s^−1^.

## 4. Conclusions

We demonstrated that RAS acts as a powerful technique to use in order to detect the first steps of porphyrin nanocrystals dissolution in acidic liquid media. By monitoring the optical anisotropy changes in situ and *in operando* conditions, we confirm that one-electron transfers, occurring at precise EC potentials during the oxidation of ZnTPP/HOPG, H_2_TPP/HOPG and Fe(TPP)Cl/HOPG films in sulfuric acid, are responsible for the early stages of structural modifications in porphyrin nanocrystals, in agreement with previous morphological investigations. Interestingly, after the oxidation of the organic film, the 3D phase of ZnTPP films looks completely amorphous, showing a null optical anisotropy signal and a melted-like morphology; conversely, the 3D phase of both H_2_TPP and Fe(TPP)Cl films preserves a certain crystallinity, which still gives rise to a peculiar anisotropy signal, representative of a post-oxidation condition. We speculate that the reason for the observed differences in the optical/morphological behavior of the three porphyrin systems could be related to details of the oxidation process of the porphin macrocycle; the identification of the molecular sites involved in the electron transfer processes will be the topic of future investigations using in situ X-ray absorption spectroscopy combined with electrochemistry measurements.

In summary, RAS can be used for real-time monitoring and, from the early stages, modifications of anisotropic organic/inorganic systems which are caused by damaging processes in oxidative/corrosive media or EC conditions.

## Sample Availability

Samples of the compounds ZnTPP, H_2_TPP, Fe(TPP)Cl are available from the authors.
